# A Rare Case of Atypical Fibroxanthoma of the Thigh in an Elderly Patient

**DOI:** 10.7759/cureus.33622

**Published:** 2023-01-10

**Authors:** Yaser Alotaibi, Mohammed F Bondagji, Abdulrahman M Alharthi, Abdullah Alharbi

**Affiliations:** 1 Department of Dermatology, King Fahad Armed Forces Hospital, Jeddah, SAU; 2 Department of Medicine and Surgery, College of Medicine, Umm Al-Qura University, Makkah, SAU; 3 Department of Pathology, King Fahad Armed Forces Hospital, Jeddah, SAU

**Keywords:** tumor markers, sun exposure, skin tumor, geriatric medicine, atypical fibroxanthoma

## Abstract

Atypical fibroxanthoma (AFX) is a rare low-grade soft tissue tumor that manifests in sun-damaged skin on the head or neck of elderly patients, although it can occur anywhere else in the body. In this case, we report the presence of AFX on the right thigh of a 70-year-old white female. Upon presentation, she complained of a painless mass on her thigh with no family history of AFX or sun exposure. The mass had previously been managed by incision and drainage, with no improvement. The patient underwent a biopsy, revealing a diagnosis of AFX, which was managed by surgical removal of the neoplasm with appropriate safety margins.

## Introduction

Atypical fibroxanthoma (AFX) is a benign dermal-based tumor with low malignant potential. Basal cell tumor, squamous cell carcinoma, pyogenic granuloma, and amelanotic melanoma make up most of the differential diagnoses of AFX. AFX usually presents as an exophytic, rapidly growing, dome-shaped nodule with a diameter of 1-2 cm. Secondary modifications include serosanguineous crusts and ulceration [1,2]. Additionally, it presents most commonly in elderly white patients and primarily in sun-exposed areas of the body, such as the head and neck. However, manifestations in other sites have been reported, including the trunk, upper extremities, shoulders, and dorsum of the hand. Trunk and limb lesions appear more prevalently among younger patients. The treatment for AFX is complete surgical excision, although Mohs micrographic surgery (MMS) and follow-up have become the model of care [3].

## Case presentation

**Figure 1:**
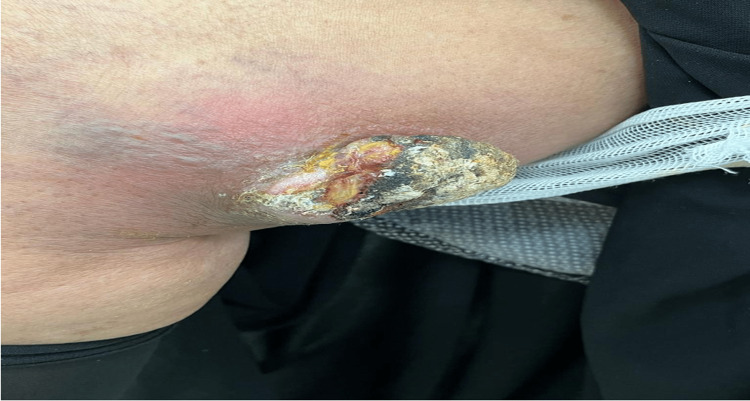
Painless mass over the medial aspect of the right thigh surrounded by erythema.

A 70-year-old female presented with a seven-month history of a skin lesion over the medial aspect of the right thigh. The lesion started as an asymptomatic, painless, solitary, and small erythematous papule with gradual growth. Prior to visiting our clinic, the patient had attended another hospital, where she had been treated with an incision and drainage of the lesion, with no improvement. Subsequently, the tumor started to increase in size again, and there was a yellowish discharge with poor healing of the lesion. The patient reported no fever, fatigue, weight loss, night sweating, or loss of appetite. She had no history of trauma, radiation therapy, insect bites, or burns. Family history was unremarkable for similar presentation or any malignancy. Physical examination revealed a well-demarcated 2 × 3 cm mass over the medial aspect of the right thigh surrounded by erythema and swelling. Two-thirds of the lesion was covered by a crust (Figure 1).

**Figure 2:**
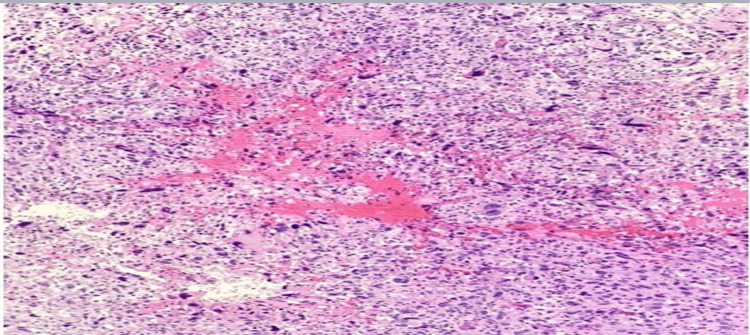
Tumor cells that are bizarre and atypical with pleomorphism in size and shape and abundant eosinophilic cytoplasm (H&E, 100×). H&E: hematoxylin and eosin

**Figure 3:**
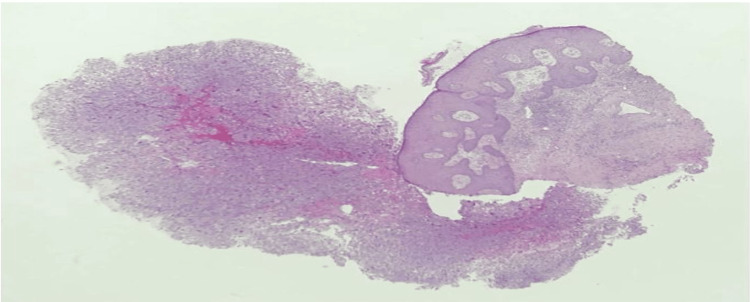
A dermal-based tumor with no connection to the overlying epidermis (H&E, 40×). H&E: hematoxylin and eosin

**Figure 4:**
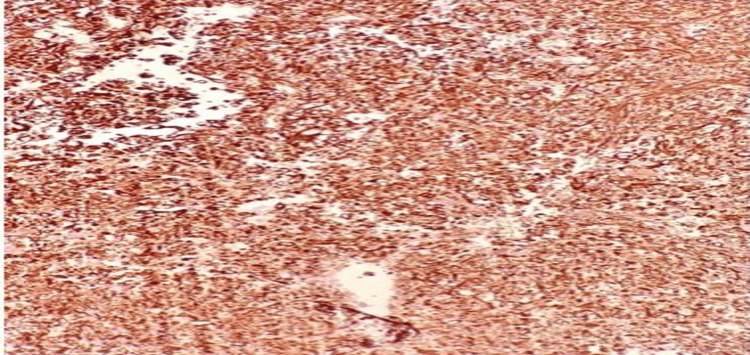
Tumor cells that are positive for vimentin.

**Figure 5:**
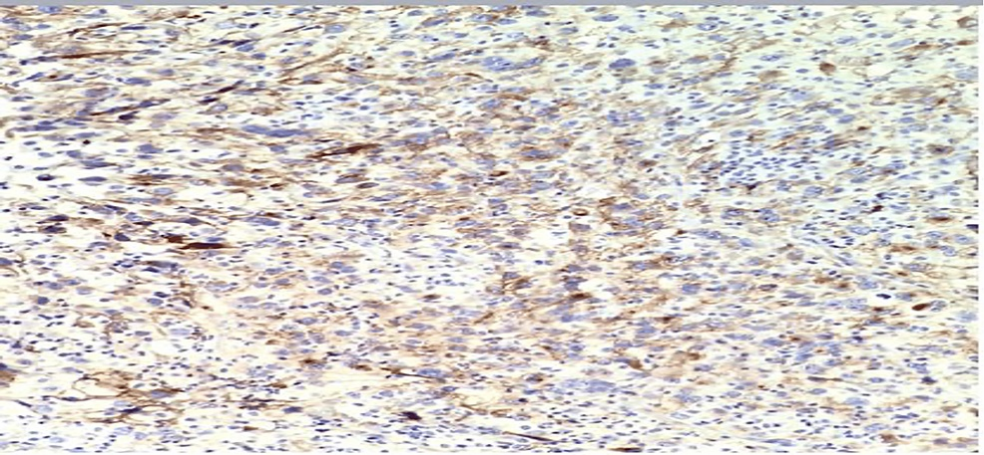
Tumor cells that are positive for CD10. CD10: cluster of differentiation 10

We performed a 4 mm skin punch biopsy for histopathology and immunohistochemistry to reach a diagnosis. The histopathology consisted of dermis and epidermis sections, and there was a dermal-based poorly differentiated pleomorphic neoplasm composed of large and bizarre spindled to epithelioid cells with abundant eosinophilic cytoplasm. There were occasional cells with pleomorphic and hyperchromatic nuclei (Figure 2), but there was no connection to the overlying epidermis, and no in situ component was seen (Figure 3). Immunohistochemistry markers of tumor cells were positive for vimentin (Figure 4) and cluster of differentiation (CD) 10 (CD10) only (Figure 5) and were negative for CD68, p63, CD20, and epithelial membrane antigen (EMA). Immunohistochemical staining was performed with all controls showing appropriate reactivity. The histological features and immunophenotyping were suggestive of AFX.

We recommended this patient undergo surgical removal of the entire tumor with MMS or wide local excision (WLE), as AFX generally has a good prognosis and rarely metastasizes. The patient was referred to the general surgery department for the surgical removal of the 2 × 3 cm size tumor with adequate safety margins of 2 cm. The patient was scheduled for a follow-up after three months with the dermatology clinic after the removal of the tumor to assess if there are signs of recurrence or any complications. Fortunately, the patient’s condition was reassuring.

## Discussion

AFX typically manifests as an ulcerated nodule in the head or neck of elderly patients with sun-damaged skin and generally grows in a contiguous pattern. Histologically, AFX consists of spindle cells mixed with bizarre, multinucleated giant cells, making the neoplasm relatively simple to recognize on frozen sections [1,2]. In comparison, a review that was conducted in 2019 states that the diagnosis of AFX and pleomorphic dermal sarcoma (PDS) neoplasms continues to be difficult and relies on the process of exclusion because these neoplasms demonstrate no consistent discriminating morphology or immunohistochemical (IHC) characters [4]. In most cases, reasonable initial IHC markers inclusive of SRY-related HMG-box 10 (SOX10) and/or S100, cytokeratins, and desmin/caldesmon will help differentiate between the most prevalent neoplasms involved in the differential diagnosis, such as melanoma, leiomyosarcoma, and squamous cell carcinoma [4]. The addition of procollagen-1 and/or CD10 might be beneficial as comparatively effective tumor markers in AFX [5]. AFX has a high risk for local recurrence with low metastatic potential and can be treated either by WLE or MMS, although no specific surgery is considered an optimal treatment [6]. However, MMS has a lower recurrence rate compared with WLE because the better results of these AFX patients are probably influenced by careful and perfect margin control through MMS. Among 175 patients managed by MMS, only five had a local recurrence (recurrence rate: 2%, 95% confidence interval (CI): 0%-4.1%), while out of 732 patients managed by WLE, 68 had a local recurrence (recurrence rate: 8.7%, 95% CI: 5%-12.3%). Five tumors removed by MMS metastasized (metastatic rate: 1.9%, 95% CI: 0.1%-3.8%), whereas 17 metastases were detected in patients with WLE (metastatic rate: 1.0%, 95% CI: 0.2%-1.9%) [6].

There are multiple factors associated with recurrence, including intrusion into subcutaneous fat, poorly circumscribed neoplasm, and insufficient or imperfect excision. Recurrence might present clinically with induration of the lesion, ulcers, or poor healing of the tumor at the site of surgical excision, and in case of recurrence or metastatic disease, it may be necessary to have radiation and/or chemotherapy as an adjuvant treatment, despite the lack of proof of effectiveness. Regarding the use of radiation therapy in AFX, there are no specific guidelines or recommendations [7]. Most instances of AFX have certain alterations in dipyrimidines in the P53 and TERT promoter genes, indicating that tumor formation is, at least in portion, induced by UV-triggered mutations [8,9].

## Conclusions

This case highlights an elderly patient who was suspected to have AFX. However, after a full history was obtained and a physical examination was done on the patient, a punch biopsy was performed on her and was sent for histopathology and immunohistochemistry, which resulted in the diagnosis of AFX. The patient was referred to the general surgery department for the surgical removal of the tumor. AFX should be treated to avoid any complications or metastasis, although this is rare. Furthermore, while commonly linked with specific sites and sun exposure, it is important to note that this tumor can present anywhere in the body whether exposed to sunlight or not.
